# High-Threshold Mechanosensitive Ion Channels Blocked by a Novel Conopeptide Mediate Pressure-Evoked Pain

**DOI:** 10.1371/journal.pone.0000515

**Published:** 2007-06-13

**Authors:** Liam J. Drew, Francois Rugiero, Paolo Cesare, Jonathan E. Gale, Bjarke Abrahamsen, Sarah Bowden, Sebastian Heinzmann, Michelle Robinson, Andreas Brust, Barbara Colless, Richard J. Lewis, John N. Wood

**Affiliations:** 1 Department of Biology, University College London, London, United Kingdom; 2 Centre for Auditory Research, University College London Ear Institute, London, United Kingdom; 3 Xenome Ltd, Indooroopilly, Queensland, Australia; 4 Institute for Molecular Bioscience and School of Biomedical Sciences, The University of Queensland, St. Lucia, Queensland, Australia; 5 Fondazione Santa Lucia, Centro Europeo di Ricerca sul Cervello, Rome, Italy; 6 Ionix Pharmaceuticals Ltd, Cambridge, United Kingdom; The Rockefeller University, United States of America

## Abstract

Little is known about the molecular basis of somatosensory mechanotransduction in mammals. We screened a library of peptide toxins for effects on mechanically activated currents in cultured dorsal root ganglion neurons. One conopeptide analogue, termed NMB-1 for noxious mechanosensation blocker 1, selectively inhibits (IC_50_ 1 µM) sustained mechanically activated currents in a subset of sensory neurons. Biotinylated NMB-1 retains activity and binds selectively to peripherin-positive nociceptive sensory neurons. The selectivity of NMB-1 was confirmed by the fact that it has no inhibitory effects on voltage-gated sodium and calcium channels, or ligand-gated channels such as acid-sensing ion channels or TRPA1 channels. Conversely, the tarantula toxin, GsMTx-4, which inhibits stretch-activated ion channels, had no effects on mechanically activated currents in sensory neurons. In behavioral assays, NMB-1 inhibits responses only to high intensity, painful mechanical stimulation and has no effects on low intensity mechanical stimulation or thermosensation. Unexpectedly, NMB-1 was found to also be an inhibitor of rapid FM1-43 loading (a measure of mechanotransduction) in cochlear hair cells. These data demonstrate that pharmacologically distinct channels respond to distinct types of mechanical stimuli and suggest that mechanically activated sustained currents underlie noxious mechanosensation. NMB-1 thus provides a novel diagnostic tool for the molecular definition of channels involved in hearing and pressure-evoked pain.

## Introduction

The molecular transduction mechanisms mediating the detection of mechanical stimuli in the somatosensory and auditory/vestibular systems remain undefined [1], [2]. The vast majority of primary somatosensory neurons are responsive to some form of mechanical stimuli [3]. Sensory ganglia contain both low threshold mechanoreceptors (LTMs), which respond to innocuous touch or internally generated movements, and high threshold nociceptors that sense noxious levels of pressure [3]. In these cells, transduction occurs at peripheral terminals by direct gating of mechanosensitive cation channels [4], [5] that await molecular characterization. Likewise in hair cells, hair bundle displacement gates mechanosensitive ion channels that are well characterized physiologically but that are of unknown molecular identity [1], [6].

Given the inaccessibility of sensory neuron peripheral terminals, cultured DRG neurons have been used to study mechanotransducing ion channels [7]–[11]. Using a glass probe to apply pressure to the cell membrane, we have identified three classes of mechanically activated currents that differ in their kinetics, magnitude and distribution. These currents are differentially expressed in subsets of sensory neurons and have properties consistent with the physiological properties of low threshold mechanoreceptors and nociceptors [9], [10].

Pharmacological studies of mechanosensitive ion channels have been dominated by the use of low affinity, non-selective drugs (particularly gadolinium, amiloride and aminoglycosides) [12]. The discovery of selective inhibitors remains highly desirable. For example, the tarantula peptide GsMTx-4 [13] inhibits stretch-activated ion channels in a number of cell types and has given insight into the function of these channels in cardiac physiology [14].

In this study, we describe the identification of a conopeptide analogue, NMB-1 (noxious mechanosensation blocker-1), that selectively inhibits slowly adapting mechanically activated currents in DRG neurons. Peptide-sensitive currents were associated with nociceptive neurons and neurons that expressed the nociceptor marker peripherin showed selective staining with a biotinylated form of the peptide. The selective inhibition of slowly adapting currents allowed us to test the behavioral consequences of blocking this type of channel. NMB-1 selectively inhibited pain behavior in response to high intensity mechanical stimuli.

Unexpectedly, NMB-1 also blocked rapid FM1-43 loading of hair cells, a process probably mediated by mechanotransducing hair cell ion channels [15], [16]. This suggests a link between the channels mediating mechanotransduction in the two systems. These findings demonstrate that pharmacologically distinct mechanosensors are present in mammalian sensory neurons, and that slowly adapting mechanosensitive channels transduce high intensity noxious pressure.

## Results

### NMB-1 is a selective antagonist of slowly adapting mechanically activated currents in sensory neurons

**Figure 1:**
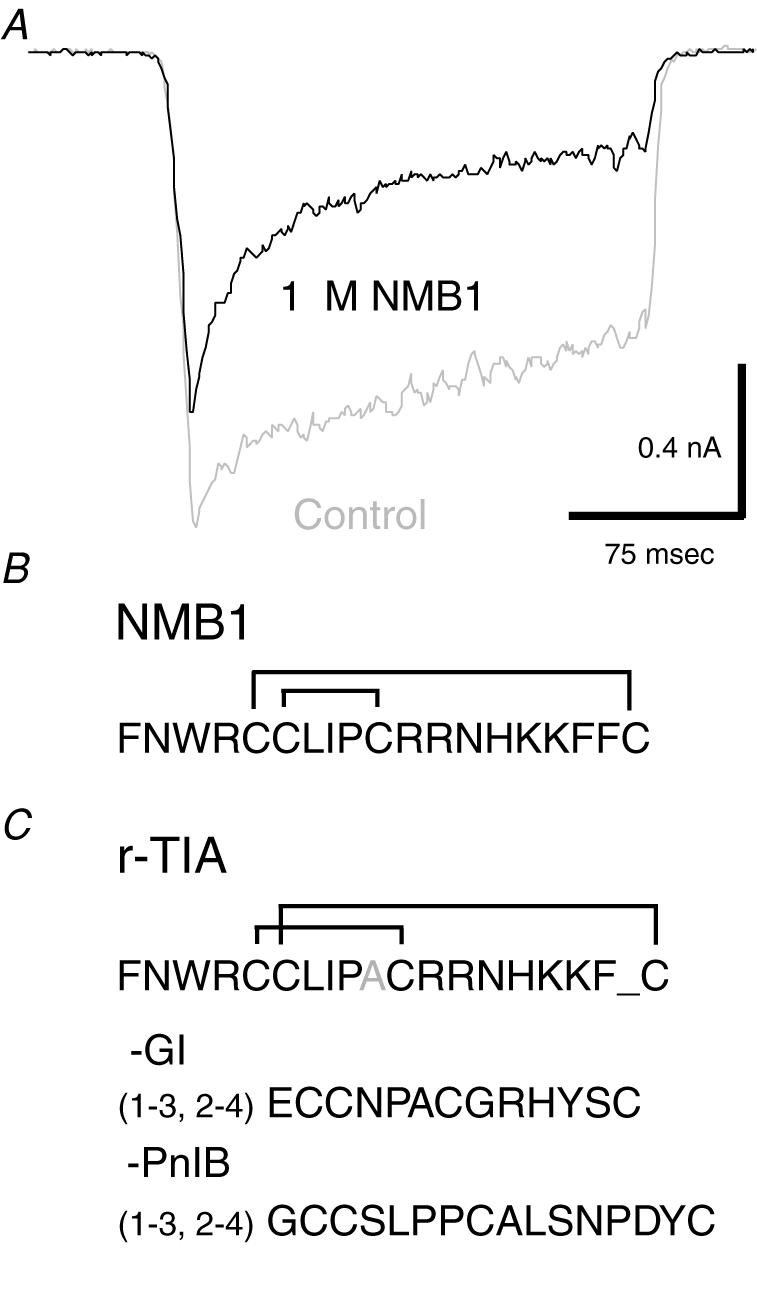
Action and sequence of the conopeptide NMB-1. (A) Inhibition of an MA current by 1 µM NMB-1. (B) Primary sequence of NMB-1 showing disulfide bridges. (C) Conopeptides closely related to NMB-1. Shown are *ρ*-TIA (from *Conus tulipa*), with differences from NMB-1 indicated, and the α-conopeptides, α-GI (*Conus geographus*) and α-PnIB (*Conus pennaceus*).

A synthetic library of >500 cloned peptide toxins and related molecules was screened for inhibitory actions on mechanically activated currents in DRG neurons. The initial screen used groups of 25 peptides at a concentration of 1 µM. Active groups were sub-divided until an individual peptide was isolated that significantly inhibited these currents (Fig. 1a). The most efficient antagonist, termed NMB-1, was identified as an analogue of ρ-TIA, a peptide originally isolated from the fish-eating, marine snail *Conus tulipa* [17]. NMB-1 is a 19 amino acid polypeptide, related to the 2-loop ρ-conotoxin class, that contains 4 cysteines linked by disulphide bridges in the [1], [4] [2], [3] configuration (Fig. 1b). NMB-1 differs from the closely related ρ-TIA (which targets α1-adrenoreceptors) by 2 amino acids and has a different disulphide bridge structure compared to ρ-TIA and the more distantly related α-conopeptides that block nicotinic acetylcholine receptors [17] (Fig. 1c). ρ-TIA itself had some antagonist activity on mechanically gated ion channels at 1 µM but the level of blockade was substantially less than seen with 1 µM NMB-1 (data not shown).

**Figure 2:**
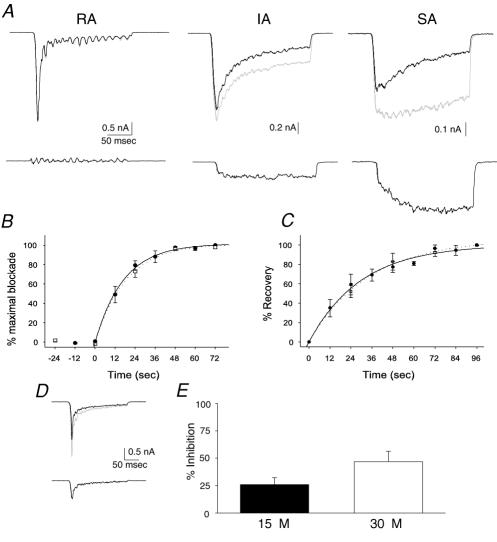
NMB-1 has 30-fold selectivity for ion channels underlying SA currents over those mediating RA currents. (A) *Upper*. Example traces showing the action of 1 µM NMB-1 on RA (*left*), IA (*center*) and SA (*right*) currents; control responses are in *gray* and currents in NMB-1 are in *black*. NMB-1 had no effect on RA currents but had significant inhibitory effects on IA and SA currents. *Lower*, Subtraction of traces recorded in 1 µM NMB-1 from control traces shows that NMB-1 selectively inhibits a persistent component of the response at this concentration. (B) The rate at which blockade by NMB-1 develops is independent of stimulation frequency (i.e. channel open time). 1 µM NMB-1 was applied to 3 neurons that were stimulated at 12 and 24 sec intervals; the association constants were 2.50×10^4^ M^−1^s^−1^ and 2.47×10^4^ M^−1^s^−1^, respectively. (C) The rate at which blockade by NMB-1 is relieved following drug removal is also independent of stimulation frequency. For 1 µM NMB-1 (n = 3) the disassociation constants were 0.034 s^−1^ (12 sec) and 0.029 s^−1^ (24 sec). (D) Example traces showing the effect of 30 µM on a RA current (*top*) and the blocked current (from subtraction, *bottom*). (E) Summary of inhibition of RA currents by 15 µM and 30 µM NMB-1 (n = 4); currents were inhibited by 25.9±6.3% and 46.8±9.5%, respectively.

We tested the effects of NMB-1 on mechanically activated currents according to their adaptation kinetics. They were classified as either rapidly (RA), intermediately (IA) or slowly (SA) adapting [13]. At a concentration of 1 µM NMB-1 significantly inhibited IA and SA currents but had no effect on RA currents (Fig. 2a). Subtraction of the residual currents from the native IA and SA currents revealed that the blocked components had slowly or non-adapting kinetics (Fig 2b). Because the peptide inhibited the persistent component of mechanically activated currents with smaller effects on peak current amplitude, we used total charge transfer (from the integral of the current waveform) rather than peak amplitude as a measure of current magnitude.

These data suggest that IA currents comprise SA and RA components and that the toxin selectively blocks the slowly adapting currents. Alternatively, NMB-1 may be an open channel blocker, with functional block increasing during the stimulus. To determine if NMB-1 could only bind or unbind open ion channels, we applied the toxin (1 µM) to neurons whilst they were stimulated at different frequencies, either every 12 sec or every 24 sec (slow channel inactivation precluded more frequent stimulation). These experiments revealed that stimulation frequency had no effect on either the on- or the off-rate of channel blockade, strongly suggesting that binding is independent of the channels being open (Fig. 2c, d). This conclusion is further supported by the histochemical observation that NMB-1 binds to a subset of unstimulated neurons (see below). Using the on and off rates derived from these experiments the apparent K_D_ of NMB-1 was calculated. The association and dissociation constants were approximately 2.5×10^4^ M^−1^s^−1^ and 0.03 s^−1^, respectively. The calculated K_D_ was 1.19 µM at 24 sec intervals and 1.36 µM at 12 sec. To determine the selectivity of NMB-1, it was applied at higher concentrations to neurons displaying RA currents; effects were somewhat variable but at 15 µM RA currents were inhibited by 25.9±6.3% and at 30 µM by 46.8±9.5% (Fig. 2e, f). These results thus show that NMB-1 has an approximate 30-fold selectivity for ion channels mediating SA currents over those mediating RA currents.

**Figure 3:**
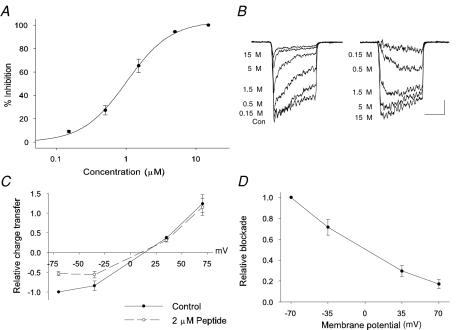
Blockade of SA currents by NMB-1 is concentration and voltage dependent. (A) Concentration-inhibition relationship for NMB-1 and SA currents; 4 neurons with SA currents were selected and 5 concentrations of NMB-1 (0.15 to 15 µM) were applied to each with full reversal in wash between application of the next concentration. The derived IC_50_ was 1.0 µM and the Hill-coefficient was 1.4. (B) Example traces of currents in each concentration of NMB-1 (*left*, as indicated) and the derived blocked current for each concentration (*right*, as indicated). (C) Current-voltage relationship for currents in control conditions (•) and in the presence of 2 µM NMB-1 (○); all values are normalized to charge transfer in control solutions at −70 mV (n = 3, mechanical stimuli applied for 100 msec). (D) Data from *C* shown as the relationship between fractional blockade by 2 µM NMB-1 and membrane potential; block declines linearly as the membrane is depolarized from −70 mV.

To further analyze the affinity of NMB-1 for the ion channels underlying SA currents, neurons were selected that displayed predominately SA currents and the peptide was applied at ascending concentrations from 0.15 to 15 µM (Fig. 3a–c). Despite the overall slow adaptation of the control currents, in all cells tested a minor component of the response was attributable to RA ion channels (Fig. 3b). Given the blockade of RA currents at higher concentrations of NMB-1, SA current blockade was normalized to the level at 15 µM; this was justified by the near saturation of blockade between 5 and 15 µM. A significant inhibitory activity was apparent at 0.15 µM and the apparent IC_50_ of NMB-1 on SA currents was found to be 1.0 µM, close to the K_D_ estimated from its association and dissociation rates. The apparent Hill coefficient was 1.4 (Fig. 3a) suggesting that there is some positive co-operativity in toxin binding.

The voltage-dependence of current block by NMB-1 was assessed by recording mechanically activated currents at four membrane potentials (−70, −35, +35 and +70 mV). In quasi-physiological solutions, currents reverse slightly positive to 0 mV with a relatively linear I-V relationship (LJD and PC unpublished observations; Fig. 3d. See also Ref. 18). Applying 2μM NMB-1 at each membrane potential revealed that channel block was strongly voltage dependent. The degree of inhibition was linearly related to membrane potential and the level of blockade at +70 mV was approximately 20% of that seen at −70 mV (Fig. 3d, e). NMB-1 has a net positive charge and these data suggest that the peptide-ion channel interaction is dependent on the electric field across the membrane.

**Figure 4:**
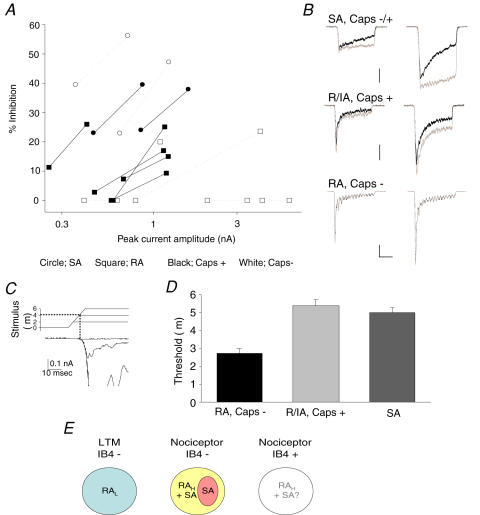
SA currents are more prevalent at high intensity mechanical stimuli; evidence from NMB-1 activity at 2 stimulus intensities and activation thresholds. (A) Blockade by 1 µM NMB-1 of currents evoked by 9 and 12 µm stimuli. Each connected pair of symbols on the graph represents a single neuron. The symbol on either end indicates the peak current amplitude and the level of inhibition of charge transfer by 1 µM NMB-1; the left-hand symbol represents the response to a 9 µm stimulus and the right-hand one the response to 12 µm. Capsaicin-sensitive neurons are represented by black symbols and solid lines, whereas capsaicin-insensitive neurons are shown by white symbols with dotted lines. SA currents are indicated by circles, and squares represent RA currents. The results show the heterogeneity of currents and the different ratios of NMB-1 sensitive and insensitive ion channels in each cell; NMB-1 has a greater inhibitory effect at the larger stimulus intensity and is most active in capsaicin-sensitive neurons and neurons with SA currents. In capsaicin-insensitive neurons with RA currents 2/5 are NMB-1 sensitive and only at a 12 µm stimulus. Only neurons with currents >250 pA were selected for these experiments. (B). Example traces of currents (Grey = control, black = 1 µM NMB-1) activated by 9 µm (*left*) and 12 µm (*right*) stimuli in a capsaicin sensitive neuron (*top*), a neuron with a SA current (*middle*) and a capsaicin insensitive neuron with a RA current (*bottom*). (C) Expanded view of a family of currents evoked by ascending mechanical steps showing that currents activate at a specific membrane displacement and how this allows extrapolation of the threshold of activation. (D) Graph shows the average activation thresholds for capsaicin insensitive neurons with RA currents (2.7±0.3 µm, n = 31) that were lower than those of neurons with SA currents (5.0±0.3 µm, *P*<0.001, n = 9) and capsaicin sensitive neurons (5.4±0.4 µm, *P*<0.001, n = 32). (E) Schematic of ion channel distribution across subpopulations of DRG neurons. Each color represents a functional class of neurons and indicated are the MS channel types expressed by that class.

We next examined which classes of DRG neurons express NMB-1-sensitive mechanically activated currents. Previous findings suggest that currents with intermediate or slow adaptation rates are associated with nociceptive neurons [13]. Using NMB-1 to distinguish RA and SA currents, we investigated the responses of nociceptors and LTMs to mechanical stimulation. We also examined the relative contribution of each current type when the stimulus magnitude was increased, i.e. are the relative proportions of current carried by NMB-1 sensitive and insensitive channels constant at different stimulus intensities? NMB-1 (1 µM) was applied to neurons mechanically stimulated at two intensities, 9 and 12 µm, which were then tested for capsaicin sensitivity (Fig. 4a). Four neurons (two of which responded to capsaicin) displayed SA currents; 1 µM NMB-1 significantly inhibited SA currents at both stimulus intensities although the level of blockade was greater at the higher level, 27.3±4.1% (9 µm) versus 45.1±4.2% (12 µm, n = 4, *P* = 0.02, Fig. 4a, b). In low threshold mechanoreceptors (i.e. capsaicin-insensitive cells displaying RA currents) the toxin did not affect currents evoked by a 9 µm stimulus although at the higher stimulus intensity 2/5 neurons displayed toxin sensitivity. Conversely, in nociceptors (capsaicin-sensitive neurons) with RA currents, the toxin was active in all cells (5/5) at the higher stimulus intensity (currents decreased by 18.5±3.2%) but also had inhibitory actions in 3/5 neurons when stimulated by a 9 µm displacement. In these capsaicin-sensitive neurons, current adaptation (over 200 msec) decreased when the stimulus was increased from 9 to 12 µm (94.4±2.1% to 83.2±3.7%, *P* = 0.03. Fig. 4a, b), consistent with a relative increase in SA current amplitude. These experiments demonstrate that most neurons co-express RA and SA mechanosensitive ion channels but that SA currents are expressed predominantly in nociceptive neurons. Moreover, in nociceptors, as the intensity of mechanical stimulation increases, NMB-1-sensitive SA currents account for a greater fraction of the mechanically evoked response.

To examine the possibility that SA currents are expressed in neurons derived from high-threshold mechanonociceptors, the activation thresholds for different current types were assessed in a larger sample of neurons (Fig. 4c). Neurons insensitive to capsaicin that expressed RA currents had low thresholds of activation (2.7±0.3 µm, n = 31), consistent with the majority of these being low threshold mechanoreceptors. Conversely, SA currents and RA currents in capsaicin-sensitive neurons activated at significantly higher thresholds, 5.0±0.3 µm (*P*<0.001, n = 9) and 5.4±0.4 µm (*P*<0.001, n = 32), respectively (Fig. 4d), consistent with the detection of noxious pressure levels.

**Figure 5:**
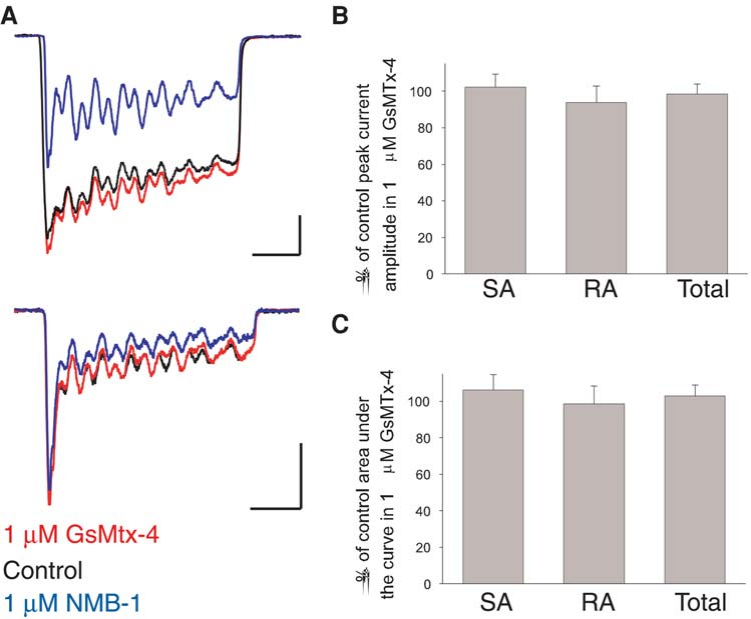
The stretch-activated channel blocker GsMTx-4 does not block mechanically activated currents in DRG neurons. (A) Example traces of SA (*top*) and RA (*bottom*) mechanically activated currents. Shown are the effects of GsMTx-4 (1 µM, *red*) and for comparison, NMB-1 (1 µM, *blue*). Scale bars: horizontal, 50 msec, vertical, 0.5 nA. GsMTx-4 did not significantly alter either the peak current amplitude (B) or the charge transfer (C) through DRG channels.

To assess the specificity of NMB-1 we tested its effects (at 2 µM) on a range of cationic currents in cultured DRG neurons. Voltage-activated sodium, calcium and potassium currents as well as capsaicin-evoked TRPV1 currents and ASIC-mediated low pH-evoked currents were recorded separately in different neurons (see Methods S1) and no significant effect of NMB-1 was observed on any of them (Table 1). We also tested the tarantula toxin GsMTx-4 on mechanically activated currents in sensory neurons; this toxin inhibits stretch-activated cation channels in cardiomyocytes [13], astrocytes and smooth and skeletal muscle cells [19] but does not inhibit mechanotransduction in the inner ear [20]. GsMTx-4 was found to be inactive (Fig. 5) confirming that ubiquitous stretch-activated ion channels do not mediate sensory neuron mechanically activated currents.

**Table 1:** NMB-1 is selective for MA currents in DRG neurons.

Parameter (2 µM)	% Change in peak	*n*
TRPV1	+7.4±8.8	4
ASIC	+2.6±2.5	3
Resting membrane potential	−0.4±0.3	5
Ca^2+^ Current	−3.9±8.1	4
K^+^ Current	−4.1±5.5	8
TTX_S_ Na^+^ Current	−3.6±8.3	3
TTX_R_ Na^+^ Current	+3.0±4.6	3

No current type recorded from DRG neurons was significantly affected by 2 µM NMB-1; neither peak amplitudes nor current kinetics (data not shown) were affected.

### NMB-1 selectively binds to a sub-population of DRG neurons

**Figure 6:**
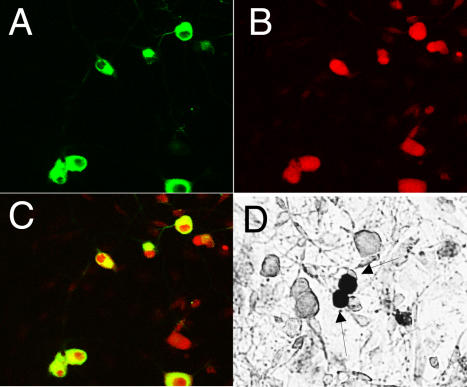
Biotinylated NMB-1 binds to peripherin-positive DRG neurons. Primary cultures of DRG neurons were stained with biotinylated NMB-1 and streptavidin linked to Cy3. (A,C) or alkaline phosphatase (D). Counter-staining with a monoclonal antibody to peripherin (B) shows in merged figure (C) that nearly all NMB-1 binding neurons express peripherin. Large diameter sensory neurons (E) were negative for NMB-1 binding. No signal was visible when NMB-1 was omitted from the staining procedure.

We characterized the sensory neurons that bind NMB-1, using a biotinylated form of the peptide and a fluorescent streptavidin derivative, together with immunocytochemical markers for sensory neuron subsets. The biotinylated derivative of NMB-1 (1μM) showed channel block indistinguishable from that observed with the unmodified peptide. We found that small diameter sensory neurons that express peripherin represented the major class of NMB-1 binding neurons (Fig 6a–c). Nearly all peripherin-positive neurons bound biotinylated NMB-1 and almost all NMB-1-positive cells showed peripherin immunoreactivity. NMB-1 binding was not observed in large diameter DRG neurons (Fig. 6d). Peripherin-expressing neurons are predominantly nociceptive, consistent with a role for NMB-1 binding ion channels in damage sensing.

### NMB-1 blocks mechanotransducing ion channels in cochlear hair cells

**Figure 7:**
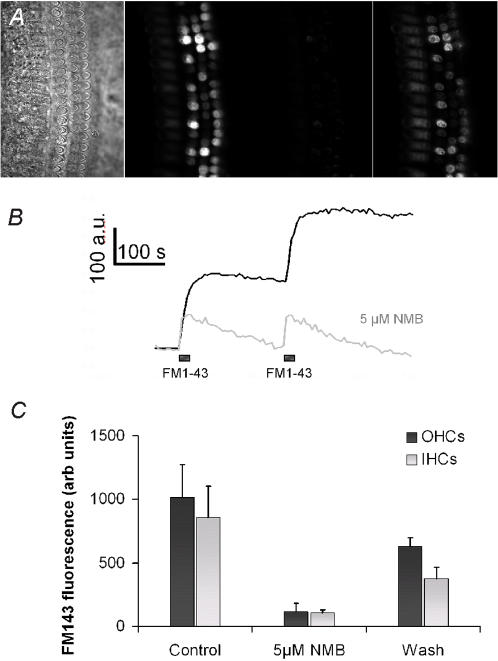
NMB-1 inhibits mechanotransduction in sensory hair cells from the rat cochlea. (A) Left panel shows DIC image of a rat cochlear culture. OHCs are clearly visible by their V-shaped sensory hair bundles. IHCs are out of focus in this view but their position is indicated for reference. FM1-43 was applied by local pipette perfusion for 10 seconds (two times). The confocal fluorescence images shown are subtracted images showing the change in FM1-43 derived fluorescence at the end of the 2×10 second applications. In the presence of 5 µM NMB-1 there is minimal FM1-43 loading. After washout FM1-43 loading recovers. (B) Time course data showing the loading of FM1-43 measure in a single outer hair cell as a function of time during the two 10 second applications. In the presence of 5 µM NMB-1 FM1-43 loading is blocked. (C) Pooled data showing the changes in FM1-43 fluorescence after 2×10 second applications from 4 different cultures (samples are averages from the same 3 cells in each experiment) for control, NMB-1 and subsequent recovery after wash out. Mechanotransduction-dependent loading of FM1-43 is blocked by 5 µM in both OHCs and IHCs.

To further assess the specificity of NMB-1, we examined its actions on mechanotransducing ion channels in cochlear hair cells. We used rapid uptake of the styryl dye FM1-43 by hair cells as an assay; uptake occurs through mechanosensitive ion channels that are 10–15% open at resting bundle positions [15], [16]. FM1-43 was applied to organotypic cochlear cultures (Fig. 7a) for two consecutive 10 second periods in each condition; in control conditions this resulted in significant and maintained uptake of the dye into the cytoplasm of both inner (IHC) and outer hair cells (OHC) (Fig. 7). When FM1-43 was applied in the presence of 5μM NMB-1, the increase in cytoplasmic fluorescence decreased by 80–85% in IHCs and OHCs (Fig. 7a–c) and displayed a transient temporal profile associated with non-specific labeling of the apical plasma membranes of these cells (Fig. 7b). Dye uptake recovered upon washout of the peptide (Fig. 7a, c). The efficacy of NMB-1 in this assay suggests that it has a similar affinity for mechanotransducing ion channels in hair cells as it does for SA ion channels in DRG neurons.

It has been suggested that TRPA1 is a component of the mechanotransducing ion channel complex in mammalian hair cells [21] however the absence of a detectable deficit in hair cell mechanotransduction in mice lacking the gene for TRPA1 argues strongly against this hypothesis [22], [23]. We found that the activation of TRPA1 expressed in CHO-K1 cells by mustard oil was not inhibited by NMB-1 (Fig. S1).

### NMB-1 inhibits behavioral responses to high intensity mechanical stimulation

**Figure 8:**
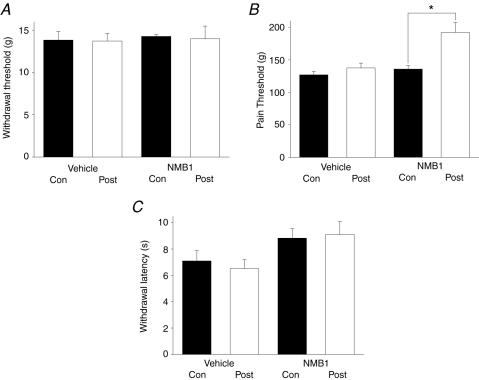
NMB-1 selectively inhibits behavioral responses to high intensity noxious mechanical stimuli. (A) The effect of NMB-1 on the 50% PWT in the von Frey test; data is shown as the percentage of post-injection values over pre-injection values. Neither NMB-1 (20 nMoles, n = 8) nor its vehicle (0.1% BSA in external solution, n = 8) had an effect on PWT. (B) NMB-1 (2×20 nMoles, n = 8, Student's unpaired t-test versus vehicle effect, *P*<0.001) increased the threshold for evoking pain behaviors in rat hind paw by 41.1±7.3% in the Randall-Selitto test. Injection of 0.1% BSA solution had no behavioral effects (n = 8). (C) NMB-1 had no effect on withdrawal latencies in the Hargreaves' test for thermal sensitivity; after injection of 20 nMoles of NMB-1 latencies were 107.5±16.6% of pre-injection control values (n = 6 in each group, Student's paired test; *P*>0.8; Student's unpaired t-test versus vehicle effect, *P*>0.4).

We tested the effects of NMB-1 on the behavioral responses of rats to low and high intensity noxious mechanical stimuli. Firstly, NMB-1 (20 nMoles, given by intradermal injection into the plantar surface of the paw) was assessed in the von Frey test. This assay measures the threshold for paw withdrawal in response to punctate mechanical stimuli applied to the plantar paw surface; in naïve animals no overt discomfort is displayed when the paw is withdrawn. Injection of NMB-1 had no significant effect on paw withdrawal thresholds (Fig. 8a). Secondly, we assessed the peptide in the Randall-Selitto test. In this test ascending pressure levels are applied to the rat's hind paw until a clear pain-related behavior was evoked (i.e. writhing or vocalization). In contrast to the von Frey test, NMB-1 (2×20 nMoles, intradermally injected on the plantar and dorsal surfaces) produced a pronounced inhibitory effect, increasing the pain threshold by 41.1±7.3% (Fig. 8b). Finally, the selectivity of NMB-1 was further examined in the Hargreaves' test, a measure of the responsiveness of an animal to a radiant heat stimulus, where it was found to be inactive (Fig. 8c).

Overall, these behavioral results show differential effects of NMB-1 in modulating responses to mechanical stimuli; the compound increases the threshold for pain behavior in response to high intensity mechanical stimuli but does not affect responses to lower level mechanical stimuli that evoke a withdrawal reflex in the absence of signs of discomfort.

## Discussion

The novel conotoxin analogue, NMB-1, is a potent and selective inhibitor of slowly but not rapidly adapting mechanically activated currents in cultured DRG neurons. NMB-1 shows approximately 30-fold selectivity for the ion channels mediating persistent over rapidly adapting responses to mechanical stimuli. At low micromolar levels, NMB-1 shows no activity at ASICs, TRPV1, TRPA1 or voltage-gated sodium, potassium or calcium channels, whilst the inhibitor of stretch-activated ion channels GsMTx-4 had no effect on sensory neuron mechanically activated currents. Together these data suggest that mechanosensitive ion channels with distinct pharmacology mediate rapidly and slowly adapting currents. To our surprise, in cochlear hair cells, 5 µM NMB-1 significantly inhibited rapid FM1-43 uptake through mechanically gated ion channels [15], [16], suggesting that components of the mechanotransduction complex in hair cells and DRG neurons may be structurally related.

The slowly adapting mechanically gated ion channels blocked by NMB-1 are predominately expressed by nociceptors. We found that biotinylated NMB-1 selectively bound to peripherin-containing DRG neurons, most of which are nociceptors. In electrophysiological experiments, we showed that SA mechanosensitive ion channels have high activation thresholds and are more important in encoding large over small mechanical displacements. In the intact animal, NMB-1 showed a selective inhibition of high rather than low intensity mechanically evoked responses in behavioural assays, supporting the view that the slowly adapting mechanosensitive channels mediate responses to painful levels of pressure.

### NMB-1 is a novel peptide inhibitor of a subclass of mechanosensory ion channels

NMB-1 is the first compound that clearly distinguishes classes of mechanosensitive ion channels expressed by sensory neurons. Previous work showed differences in the mechanosensory properties of distinct classes of DRG neurons but antagonists showed no functional differences [9]. In neonatal rat neurons, capsaicin-insensitive neurons had substantially larger mechanically activated currents than nociceptive, capsaicin-sensitive neurons [10] and here we confirmed that mechanically gated ion channels in nociceptors have higher activation thresholds than in capsaicin insensitive neurons. In adult mouse neurons [10] a striking difference in the kinetics of mechanically activated currents between nociceptors and LTM neurons (distinguished by action potential duration) was observed; LTMs displayed rapidly adapting currents whereas nociceptors generated either SA currents or intermediately adapting currents (that were clearly distinct from SA and RA currents). This distribution is consistent with the present data showing that toxin-sensitive SA channels are preferentially expressed in nociceptors. We now provide evidence that SA currents have higher activation thresholds and mediate a larger component of currents evoked by higher stimulus intensities.

Selective inhibition of SA currents by NMB-1 could arise from binding to open channels, but three findings strongly argue against this. Firstly, in the majority of capsaicin-insensitive neurons that displayed RA currents, the persistent components of these responses were not affected by NMB-1. Secondly, when neurons were stimulated with different inter-stimulus intervals, peptide binding and unbinding rates were independent of the stimulus frequency. This suggests that the rate limiting binding step was independent of ion channel activity. Thirdly, biotinylated NMB-1 bound to unstimulated peripherin-positive DRG neurons, with a pattern similar to the distribution of the SA currents. (NMB-1 had no effect on resting membrane potential suggesting SA mechanosensitive channels are not open at resting membrane tensions). Interestingly, as the concentration of NMB was increased the kinetics of the blocked currents changed in a way that suggests that the peptide whilst able to actively bind the closed configuration, may have a higher affinity for the open configuration. Peptide activity was strongly voltage-dependent suggesting that NMB-1 may be a pore-blocking compound, binding within the membrane's electric field.

Given the selectivity of NMB-1 for SA currents in DRG it was surprising to find that it also inhibited rapid FM1-43 uptake by cochlear hair cells; a process mediated by the mechanotransducing ion channels of these cells. The effects of 5μM NMB-1 on the transduction channel in hair cells makes it amongst the most high affinity antagonists for this channel and it may be more selective than other known blockers. The actions of NMB-1 on currents evoked by bundle displacement will be of interest. Current pharmacological data demonstrate that mechanosensitive ion channels in DRG neurons and hair cells are different (for example, concentrations of gentamicin and amiloride that inhibit hair cell channels are inactive in the DRG). NMB-1 is therefore, either active at more than one type of mechanosensitive ion channel or at a common ion channel subunit that binds NMB-1 in different heteromeric channels in both cell types.

### NMB-1 selectively inhibits behavioral responses to high intensity mechanical stimuli

We tested NMB-1 in two behavioral tests of mechanically evoked nociception. These tests were the Randall-Sellitto assay, which measures responses to intense tissue compression and has as a threshold overt pain behavior (i.e. writhing or vocalization), and the von Frey test, which determines the withdrawal threshold to punctate stimuli. Interestingly, NMB-1 inhibited pain thresholds in the Randall-Sellitto test, but was inactive in the von Frey test. These results contrast with the behavioral effects of FM1-43, which is a permeant blocker of all mechanically activated current types in DRG neurons and increases thresholds in both of these assays [24].

Taken together, these results indicate that different mechanosensitive ion channels expressed by DRG neurons have distinct physiological functions. Our data is consistent with high-threshold SA channels in nociceptive neurons functioning primarily in response to high intensity painful stimuli whereas lower level stimuli are associated with activation of RA currents. The pathways mediating responses to von Frey and Randall-Sellito stimuli may be quite distinct; the stimulus required to reach the designated threshold behavior is 1–2 orders of magnitude greater in the latter test. In addition, when nociceptors are ablated by neonatal capsaicin or resiniferatoxin administration, animals tolerate significantly higher levels of pressure in the Randall-Sellitto assay [25], [26] but do not display deficits in the von Frey test [27]. These data suggest that withdrawal from von Frey hairs may be an escape response to a potentially tissue-damaging stimulus, which depends upon activation of signaling pathways distinct from those that evoke pain responses in the Randall-Sellitto test. It seems likely that these two tests measure distinct aspects of mechanosensation, which employ different primary transduction mechanisms.

### Multiple Mechanosensitive ion channel subtypes underlie somatic mechanosensation

This study has shown that at least two classes of mechanosensitive ion channel are differentially expressed by subsets of sensory neurons. Current types are distinguished by their activation threshold, their kinetic properties and their sensitivity to inhibition by external Ca^2+^, FM1-43 and in particular NMB-1 [9], [10], [24]. A recent study has also reported similar classes of kinetically distinct currents upon mechanical stimulation of the neurites of cultured sensory neurons [11]. It remains to be determined if the same ion channels mediate RA currents in nociceptors and low threshold mechanoreceptors. Modulation of RA currents by external Ca^2+^ is distinct in capsaicin-sensitive and insensitive neurons [9] and the mechanical thresholds of the two cell types are different, implying that distinct ion channels may mediate RA currents in nociceptors and non-nociceptors. The kinetics of channel closing in the presence of sustained pressure will have important implications for the encoding of different aspects of the stimulus, for example the magnitude and the rate of change of pressure. As a consequence, the distinct properties of the different ion channels expressed across sub-populations of sensory neurons may allow them to encode different properties of the mechanical stimulus.

Investigations into the molecular nature of mammalian mechanotransducing proteins have focused primarily on the DEG/ENaC and TRP ion channel families due to their established roles in invertebrate mechanosensation [1], [28]. Results in mammals have, however, been equivocal. Studies of the pharmacology and ionic permeability of the channels underlying whole-cell mechanically activated currents in DRG neurons [9], [10], [18] are consistent with these channels belonging to the TRP channel family. In addition, properties of the hair cell transduction channel are also similar to those of TRP channels [29]. Although a number of mammalian TRP channels have been shown to be mechanosensitive [30], [31], it remains to be determined which are operational in mammalian primary mechanosensory systems.

The inactivity of NMB-1 at TRPV1, TRPA1 and ASICs is evidence that these channels do not underlie the sustained mechanically activated currents in sensory neurons or transduce mechanical events in the ear. We also demonstrated that the tarantula toxin, GsMTx-4 does not inhibit mechanically activated currents in DRG neurons. GsMTx-4 blocks stretch-activated cation channels in astrocytes, cardiac cells and smooth and skeletal muscle cells [18] but has no effect on the mechanotransducing channels of hair cells [19]. Recent studies show that GsMTx-4 inhibits the mechanosensitive TRP channels TRPC1 [32] and TRPC6 [33] suggesting that these channels are not involved in somatosensory or auditory mechanotransduction.

The identities of the channels underlying touch and pressure-evoked pain, as well as hearing have remained elusive. NMB-1 provides the first selective pharmacological probe for a subset of mechanosensitive ion channels in sensory neurons, combined with genetic studies, this reagent should provide useful information on the molecular basis of pressure-evoked pain and hearing transduction.

## Materials and Methods

### Cell Culture

Cells were cultured from neonatal rats (P1–2) as previously described [9]. Cultures were plated on poly-L-lysine and laminin and maintained in NGF. Recordings were made 16–36 hours after plating.

### Electrophysiology, Mechanical Stimulation and Solutions

For experiments on mechanically activated currents we used previously described methods [9], [10]. Perforated patch recordings were made using an Axopatch 200B amplifier controlled by Clampex 9 (Axon Instruments). Voltage-clamp recordings were made at a holding potential of −70 mV unless otherwise stated. Mechanical stimulation of neuronal somata was with a heat-polished glass pipette controlled by a piezo-electric crystal drive (Burleigh) as described previously [9], [10]. NMB-1 (500 µM stock in standard external solution containing 0.1% bovine serum albumin (BSA)) was always applied in 0.1% BSA which had no effect on MA currents (data not shown). GsMTx-4 stocks were 100 mM. Methods S1 includes details of how currents listed in Table 1 were activated.

### Peptide Library Synthesis and Screening

The peptide library used in this study was composed of purified synthetic venom peptides. Novel sequences were obtained form cone snail venom cDNA libraries using PCR approaches. Pools of peptides were constructed from equimolar additions of 25 peptides per pool. The library is the property of Xenome Ltd and available for collaborative screening initiatives at therapeutic targets. Included in the library were single residue analogues of NMB-1 and the closely related TIA, including an alanine scan of TIA.

Peptides were synthesized on an Advanced ChemTech (ACT-396) automated peptide synthesizer as described in Methods S1. Biotinylated NMB-1 was synthesized using orthogonal Cys(Acm)/Cys(Trt) protection and biotinylation completed on the resin. Fmoc deprotected peptidyl-resin (340 mg SV = 0.152 mmol/g) was coupled with biotin (244 mg, 1 mmol) using HBTU/DIEA (1 mmol) activation in DMF (2ml/DMSO 0.8 ml). The coupling was completed after 30 min as confirmed by a ninhydrin test.

The library was divided into 21 pools, each containing 25 individual peptides. Each pool was tested for activity on mechanically activated currents expressed by DRG neurons. Pools were applied to 6–8 neurons. Of which 3–5 displayed intermediately or slowly adapting currents. Change in peak current magnitude and total charge transfer were plotted as a percentage of control value. The most active pool was selected for the second round of screening, subdivided into pools of 5 peptides and then into individual peptides. Subsequently, peptides closely related to NMB-1 were screened individually but NMB-1 produced the highest level of blockade.

### Binding of Biotinylated Conotoxin to adult rat DRG Neurons in vitro

Dissociated adult rat DRG cultures were rinsed with DMEM at room temperature and then DMEM with or without 2 µM biotinylated NMB-1 was applied for 2 minutes at room temperature. The cells were then fixed in cold 4% paraformaldehyde. Biotinylated NMB-1 was visualized by incubating cultures in alkaline phosphatase-conjugated streptavidin (Chemicon); alkaline phosphatase colour reaction was carried out using BCIP/NBT kit from Vector Laboratories according to the manufacturer's instructions. Levamisole Solution (Vector Laboratories) was added to inhibit endogenous alkaline phosphatase activity. The appearance of blue reaction product precipitate was monitored by light microscopy. To double label neurons for biotinylated NMB-1 binding and peripherin immunoreactivity, fixed cells were stained using a mouse anti-peripherin antibody (diluted 1∶600) visualized using a secondary antibody (goat anti-mouse) conjugated to the fluorescent dye Alexa Fluor 488. After washing, NMB-1 was detected by incubation in streptavidin-Cy3 solution. Pictures of the fluorescently labelled DRG neurons were taken with a Leica LSM confocal microscope (Leica Microsystems, Germany). Brightfield pictures of alkaline phosphatase stained cells were taken with an upright Zeiss microscope.

### Cochlea cultures and imaging

Cochleae were dissected from postnatal day 3–4 Sprague-Dawley rat pups in cold HEPES-buffered (10 mM, pH 7.3) HBSS (HBHBSS) as previously described for mouse [15]. Stria vascularis was removed and the cochleae were cut into basal/middle coils and placed onto MatTek® dishes coated with 50µg/ml CellTak® (Collaborative Biomedical Products, Bedford, MA, USA). Cultures were incubated in DMEM/F12 maintained at 37°C for 2 days prior to experimentation. During experiments, cultures were maintained in HEPES-buffered HBSS with 1mM Ca^2+^ and 6mM D-glucose (pH 7.3). FM1-43 (3 µM) was dissolved in HB-HBSS and was locally applied to cochlear cultures for 2×10 seconds. Images were acquired using a Zeiss 510 LSM confocal inverted microscope (excitation 488 nm, LP 560 emission). Image time series were obtained using 2 focal planes (apical pole and cell base) effective sampling rates were approximately 1 frame per second. FM1-43 was applied by local perfusion from a micropipette using a picospritzer. FM1-43 was applied (three consecutive times) for 10 seconds to minimize uptake via endocytic mechanisms. FM-143 loading was quantified by measuring the change in FM143 fluorescence by subtracting images before from those after the three applications. Measurements were made on the same areas of the culture and quantification was performed on the same cells.

### Behavioral Testing

Male C57/black mice (6–8 week, 20–25 g) and Sprague-Dawley rats (4–5 week, 120–150 g) were used for behavioral experiments. The experimenter was blind to the treatment given to each animal. All experimental procedures were carried out according to the U.K. Animals (Scientific Procedures) Act 1986.

For von Frey testing, the 50% paw withdrawal threshold (PWT) was estimated using the “up-down” method [36, 37] with von Frey hairs applied to the center of the left hind paw. Animals were habituated to the testing conditions on Day 1, exposed to the test stimuli on Day 2 and tested on Day 3. A series of stimuli were applied and then, with the animals restrained, NMB-1/vehicle (in 15 µl) was injected into the intradermally into plantar paw surface. After a period of re-settling a second series of stimuli were applied (≈20–40 minutes after injection).

For Randall-Selitto testing, an Analgesy Meter (Ugo Basile, Switzerland) was used to apply pressure to the hind paw of the animal. Rats were handled for 2 days, habituated to the Analgesy Meter for 2 days and tested on Day 5. Three pain threshold readings were taken prior to the rats being lightly anaesthetized (2–4% halothane) and NMB-1/vehicle (in 40 µl) being injected intradermally on either side of the paw. Animals were given ≈30 minutes to recover and then retested. The point where there was a clear writhing or vocalization response was taken as the pain threshold.

### Data Analysis

Data were analyzed using Clampfit 9, Sigmaplot 8 and Sigmastat 4 software. Total charge transfer was determined by integrating the current waveform. Groups of data were compared using the Student's unpaired t-test, unless otherwise stated. Data are presented as mean±SEM and a *P* value of 0.05 was taken as significant. Current adaptation was measured as the percentage decrease in current amplitude over the course of the stimulus plateau; currents were classified as slowly adapting if the current amplitude at the end of the stimulus was >75% of the peak amplitude.

## Supporting Information

Methods S1 (0.02 MB DOC)

Figure S1 NMB-1 effects on TRPA1. Lack of effect of NMB-1 on mustard oil (MO)-evoked currents in TRPA1-transfected CHO-K1 cells. (A) Note that the current does not reactivate after wash of NMB-1 by MO, indicating that the exponential decay of the current is due to desensitization rather than block by NMB-1. (B) A mix of MO and NMB-1 does not prevent activation of TRPA1 current. (A) and (B) are from two different cells. In both [MO]  = 100 µM and [NMB-1] = 2 µM. Experiments performed using an external solution containing 2mM Ca^2+^ (n = 4). Similar data were obtained using either a Ca^2+^-free external solution (n = 3) in CHO-K1 cells or a 2mM Ca^2+^-containing external solution in ND-C cells (n = 3).
(0.08 MB TIF)
