# Supplementary material for: High-Threshold Mechanosensitive Ion Channels Blocked by a Novel Conopeptide Mediate Pressure-Evoked Pain
Source: PLoS One. 2007 Jun 13;2(6):e515. doi: 10.1371/journal.pone.0000515 (PMC1885214; doi:10.1371/journal.pone.0000515)
Supplement: Methods S1 — (0.02 MB DOC) [file pone.0000515.s001.doc]

# Methods S1

# Peptide Synthesis

Peptides were synthesized on an Advanced ChemTech (ACT-396) automated peptide synthesizer on Rink amide resin using HBTU *in situ* activation protocols to couple standard Fmoc-protected amino acid to the resin. Peptides were cleaved from the resin at room temperature in TFA:H_2_O:TIPS:EDT (87.5:5:5:2.5) for 3 hr. Cold was then added to the filtered cleavage mixture and the peptide precipitated out. The diethyl ether precipitated peptides were centrifuged and washed with further cold diethyl ether to remove scavengers. The final product was dissolved in 50% aqueous acetonitrile and lyophilized. Pure, reduced peptides (1 mg/ml) were oxidized by stirring at RT in 30% DMSO/ 0.1M NH_4_OAc at pH 6.3 for ~ 24h. The solutions were diluted (DMSO was < 5%) prior to RP-HPLC purification and lyophilization.

NMB1 (FNWRCCLIPCRRNHKKFFC; 1–4,2–3 isomer) was selectively synthesized using Cys(Trt) and orthogonal Cys(Acm) of cysteines 2 and 3. After cleavage and purification of the reduced peptide the first disulfide bond was formed (1–4) in 30% DMSO/0.1M NH_4_OAc buffer. The second disulfide bond was formed by dissolving the purified, single oxidized (Acm)_2_-peptides at a concentration of 1 mM in 80% acetic acid/water. Iodine (10 eq.) dissolved in a small volume of ethyl acetate was added and the mixture stirred at RT for ~ 60 min before being quenched by addition of ascorbic acid solution (10 mg/ml). After a 5-fold dilution and adjustment to pH3, the fully oxidized NMB1 was HPLC purified.

The crude reduced and oxidized peptides were purified by HPLC (Shimadzu LC8A) using a semi-preparative C-18 HPLC column (Vydac 25cm x 10 mm) at a flow rate of 5 ml/min with a 1% gradient of 0–40% acetonitrile/0.05% trifluoracetic acid. The purity of the final product was evaluated by analytical HPLC (Zorbax 300SB C18: 4.6 x 100mm). Reduced and oxidized peptide and oxidations were monitored by electrospray mass spectrometry (Applied Biosystems API-150) operating in the positive ion mode over 300–1800 amu.

To confirm the stability of NMB-1, a 1mg/mL solution in external buffer was monitored by LC-MS. No evidence of disulfide exchange leading to isomerization was detected at 0, 1, 4 or 60 h.

***Recording of Non-Mechanically Activated Currents.***

To record voltage-activated currents, neurons were held at –70 mV and a series of 50 msec depolarizing pulses were applied in 10 mV increments to +70 mV. Below are solutions used for isolating sodium, potassium and calcium currents. All values are in mM. For potassium currents, standard potassium based internal solution was used (110 MetSO4, 30 KCl, 1 MgCl2, 10 HEPES, pH 7.35 adjusted using KOH, final K^+^ concentration 140) and the external solution was 140 NMDG, 4 KCl, 2 CaCl_2_, 1 MgCl_2_, 10 HEPES, 10 TEA-Cl, 0.5 CdCl (pH 7.35 with CsOH). For calcium and sodium currents a caesium-based internal solution was used: 110 CsMetSO_4_, 30 CsCl, 1 MgCl_2_, 10 HEPES (pH 7.35 with CsOH). The external solution for calcium currents was: 43 NMDG, 97 TEA-Cl, 4 KCl, 2 CaCl_2_, 1 MgCl_2_, 10 HEPES, 2 CsCl (pH 7.35 with HCl). For sodium currents the external solution contained 43 NaCl, 97 TEA-Cl, 4 KCl, 2 CaCl_2_, 1 MgCl_2_, 10 HEPES, 2 CsCl, 0.5 CdCl (pH 7.35 with CsOH). To record TTX-resistant currents 1 μM tetradotoxin was added.

To activate TRPV1 and ASICs, capsaicin (1 μM) and pH 6.3 (MES buffered) external solution were, respectively, applied using a Biologic rapid solution changer. Each application of capsaicin was for 10 sec whereas pH 6.3 was applied for 4 sec. For ASIC-mediated currents neurons were selected that displayed no apparent activation of TRPV1 by acidification (based on the kinetic profile of the current). Neurons were held at –70 mV, standard internal and external solutions were used see Refs. 13, 14.
