# Supplementary figures and images for: High-Threshold Mechanosensitive Ion Channels Blocked by a Novel Conopeptide Mediate Pressure-Evoked Pain
Source: PLoS One. 2007 Jun 13;2(6):e515. doi: 10.1371/journal.pone.0000515 (PMC1885214; doi:10.1371/journal.pone.0000515)

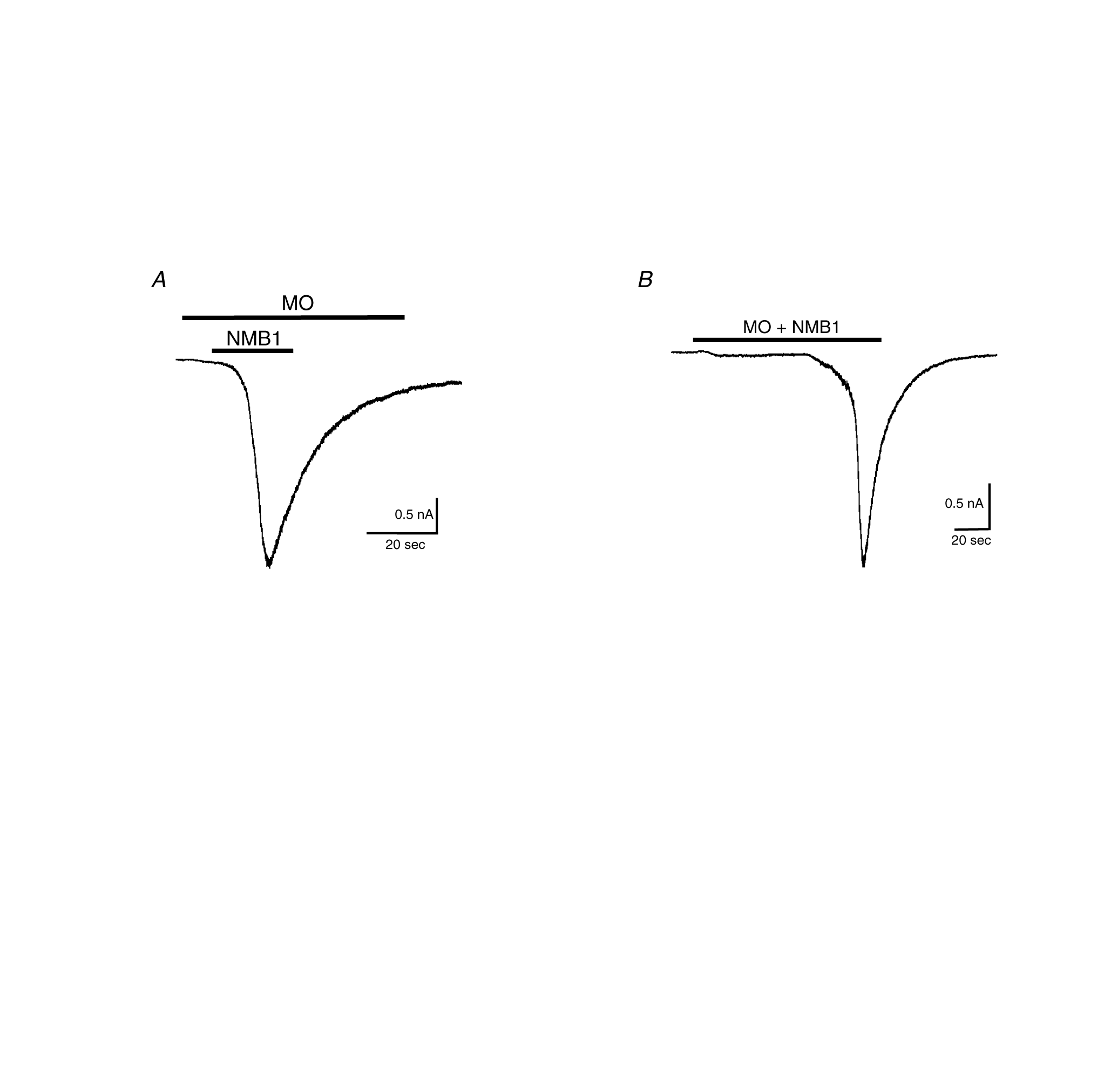

Supplement: Figure S1 — NMB-1 effects on TRPA1. Lack of effect of NMB-1 on mustard oil (MO)-evoked currents in TRPA1-transfected CHO-K1 cells. (A) Note that the current does not reactivate after wash of NMB-1 by MO, indicating that the exponential decay of the current is due to desensitization rather than block by NMB-1. (B) A mix of MO and NMB-1 does not prevent activation of TRPA1 current. (A) and (B) are from two different cells. In both [MO] = 100 µM and [NMB-1] = 2 µM. Experiments performed using an external solution containing 2mM Ca2+ (n = 4). Similar data were obtained using either a Ca2+-free external solution (n = 3) in CHO-K1 cells or a 2mM Ca2+-containing external solution in ND-C cells (n = 3). (0.08 MB TIF) [file pone.0000515.s002.tif]
